# Nitric Oxide Facilitates Delivery and Mediates Improved Outcome of Autologous Bone Marrow Mononuclear Cells in a Rodent Stroke Model

**DOI:** 10.1371/journal.pone.0032793

**Published:** 2012-03-07

**Authors:** Mallikarjunarao Kasam, Bing Yang, Roger Strong, Krystal Schaar, Vivek Misra, Xiaopei Xi, James C. Grotta, Jaroslaw Aronowski, Sean I. Savitz

**Affiliations:** Department of Neurology, Stroke Team, The University of Texas-Houston Medical School, Houston, Texas, United States of America; Julius-Maximilians-Universität Würzburg, Germany

## Abstract

**Background:**

Bone marrow mononuclear cells (MNC) represent an investigational treatment for stroke. The objective of this study was to determine the relevance of vasoactive mediators, generated in response to MNC injection, as factors regulating cerebral perfusion (CP), the biodistribution of MNC, and outcome in stroke.

**Methods:**

Long Evans rats underwent transient middle cerebral artery occlusion. MNC were extracted from the bone marrow at 22 hrs and injected via the internal carotid artery or the femoral vein 2 hours later. CP was measured with MRI or continuous laser Doppler flowmetry. Serum samples were collected to measure vasoactive mediators. Animals were treated with the Nitric Oxide (NO) inhibitor, L-NAME, to establish the relevance of NO-signaling to the effect of MNC. Lesion size, MNC biodistribution, and neurological deficits were assessed.

**Results:**

CP transiently increased in the peri-infarct region within 30 min after injecting MNC compared to saline or fibroblast control. This CP increase corresponded temporarily to serum NO elevation and was abolished by L-NAME. Pre-treatment with L-NAME reduced brain penetration of MNC and prevented MNC from reducing infarct lesion size and neurological deficits.

**Conclusions:**

NO generation in response to MNC may represent a mechanism underlying how MNC enter the brain, reduce lesion size, and improve outcome in ischemic stroke.

## Introduction

Experimental findings suggest various stem cells and other types of cells may reduce brain damage caused by ischemic injury in animal stroke models [1]–[3]. Cells originating in bone marrow are one of many different cellular sources that have been shown to improve outcome in animal models of middle cerebral artery occlusion (MCAO). Mononuclear cells (MNCs) for autologous transplantation can be isolated within hours of bone marrow aspiration for immediate use unlike mesenchymal stem cells that require several weeks of growth in culture for purification.

MNCs improve outcome in animal models of cerebral ischemia [4]–[6] possibly by exerting cytoprotective effects and reducing infarct maturation [4]. Intravenous (IV) and intra-arterial (IA) injection have been shown to deliver MNCs to the ischemic brain; however, only a fraction of injected cells migrate to the brain while the rest spread to other organs [6]. Increased number of MNCs in the brain after stroke may contribute to better recovery. Thus more information about the mechanisms governing the deposition and biodistribution of these cells after intravascular injection may help to optimize brain delivery of these cells and recovery after stroke. In this study, we investigated the effects of intravascular injections of MNCs on cerebral perfusion (CP) and found that MNC injection transiently increases CP mediated, in part, by nitric oxide (NO). We then assessed whether changes in perfusion associated with NO affects the biodistribution of MNCs in the brain and other organs. To determine whether changes in perfusion or MNC delivery to the brain are functionally relevant, we investigated whether inhibiting NO before MNC administration would interfere with the ability of MNCs to promote tissue protection and neurological improvement in the rodent stroke model.

## Materials and Methods

### Ethics Statement

All procedures were approved by the University of Texas-Houston Health Science Center Animal Welfare Committee (Protocol Number: HSC-AWC-08-103). All surgeries were performed under isoflurane (2%) anesthesia, and all efforts were made to minimize any discomfort.

### Animals

For this study, 116 Male Long Evans (0.6–0.8 kg) retired breeder (10 month old) rats were used. All animals were double housed with free access to food and water. Subjects were maintained on a standard 12∶12hrs light/dark cycle. Table 1 provides a summary of the different experiments in this report. For all experiments, animals were randomized to treatment groups and outcome assessments were completed blinded to treatment allocations.

**Table 1:** Experimental design.

Experiments	Groups					
**MR perfusion (n = 4 per group) at 24 hrs after stroke**	Saline IV	MNCs IV	MNCs IA			
**Laser Doppler Flow (n = 6 per group) at 24 hrs after stroke**	Saline IV	MNCs IV	MNCs IA	FBCs IV		
**Levels of NO and other vasodilators (n = 5 per group) at 24 hrs after stroke**	Saline IV	MNCs IA	MNCs IV	Saline IP+SalineIV(only NO)	Saline IP+MNCs IV(only NO)	L-NAME IP+MNCs IV(only NO)
**Biodistribution of MNCs (n = 3 per group) at 1 hour after MNC injection**	Saline IP+MNCs IV	L-NAME IP+MNCs IV				
**Lesion Size (n = 8 per group) at 28 days after stroke**	Saline IP+Saline IV	Saline IP+MNCs IV	L-NAME IP+MNCs IV	Saline IV	NONOate IV	
**Functional recovery (n = 8 per group) at 1, 3, 7, 14, 21, and 28 days after stroke**	Saline IP+Saline IV	Saline IP+MNCs IV	L-NAME IP+MNCs IV	Saline IV	NONOate IV	

### Stroke Model

Reversible focal ischemia of 180 min was induced by left common carotid artery (CCA) and middle cerebral artery (MCA) occlusion as previously described [4], [7]. CP was monitored by Laser Doppler Flowmetry (LDF) over the ischemic area [7]. Rectal temperature was monitored and maintained at 37±1°C during ischemia and for the first hour of reperfusion using a feed-forward temperature controller (YSI Model 72, Yellow Springs, OH) that utilizes a heat lamp and warming blanket.

### Delivery Routes

IA injection was performed through the internal carotid artery as previously described [4]. For IV delivery, the right femoral vein was cannulated.

### Bone Marrow Harvest

Twenty-two hours after stroke, bone marrow was aspirated as described previously [4]. MNC were separated from bone marrow using Ficoll density [4]. After separation, MNC were resuspended in 1 ml of saline at 10 million cells/ml for injection.

### LDF Monitoring

Twenty-four hours after stroke, rats were anesthetized. To monitor CP, the LDF probe was placed 6 mm posterior and 5 mm lateral to bregma, which overlies the peri-infarct region [7]. The average regional CP in 20 minutes before injection was regarded as baseline and monitored continuously during the injection and 30 min afterwards. The percent change from baseline was calculated during injection and 1, 3, 5, 10, 15, 20, and 30 min post injection.

### Preparation of MNC supernatant

MNCs were collected as described elsewhere [4]. MNCs were divided and seeded with a concentration of 10 million cells/ml in Dulbecco's Modified Eagle Medium (DMEM) (GIBCO, Invitrogen, USA)+10% fetal bovine serum (FBS). All cells were incubated at 37°C. Medium was collected at different time points for measurements of vasoactive mediators. Samples from 3 animals were used in triplicate.

### Fibroblast cell preparation

Rat fibroblast cells (FBCs) (CRL-1764, ATCC, USA) were cultured and isolated as described elsewhere [8]. Briefly, one vial of cells (∼1×10^6^) was cultured in a 75 cm^2^ flask in high glucose DMEM (GIBCO, Invitrogen, USA), supplemented with 10% FBS (Hyclone Laboratories, USA), 100 unit/ml penicillin, in a humidified 5% CO_2_ incubator at 37°C with medium routinely changed every three days. When cells were 95% confluent, they were trypsinized with 0.25% trypsin+0.05% EDTA and resuspended in culture medium and counted. The cells were extensively washed and 10 million/ml cells were re-suspended in saline for treatment.

### Preparation of blood serum

At different time points - before injection, during injection, and at 0, 1, 5, 10, 15, 30, 45 and 60 min after injection, 0.3 ml whole blood was harvested from the left external jugular vein. Samples from 5 animals were used in triplicate.

### Magnetic Resonance Imaging

Scans were performed on a 7T Bruker Biospec horizontal bore scanner (USR70/30; Bruker, Germany) with a S116 gradient. Animals were maintained on 2–2.5% isoflurane during the scan and body temperature was maintained with a feedback-controlled warm air system (SA Instruments, USA). The respiration, rectal temperature, oxygen level, and heart rate were continuously monitored. Images were acquired with dual-echo rapid acquisition and relaxation enhancement (RARE) sequence [9]. MR contrast was injected through femoral vein during the acquisition of dynamic susceptibility contrast-enhanced perfusion weighted imaging (DSC-PWI). Time to peak (TTP) maps were calculated from the perfusion weighted imaging (PWI) data using BioMAP Software (Bruker, Germany) and NIH's Image J software. The diffusion weighted imaging (DWI) maps were superimposed on the PWI maps by co-registering the two images. Five regions of interests (ROIs) were placed at the boundary just outside the lesion identified as the demarcated area that had restricted diffusion on DWI. This area just outside the DWI lesion was considered the peri-infarct region. Image slices were selected randomly and the mean TTP of the ROIs was reported. MRI was performed immediately before MNC infusion and repeated at 5 minutes after infusion of MNCs.

### Vasoactive Mediators

NO was assessed by measuring nitrite level using the Griess Reagent method (Biotium, USA). 150 µl of supernatant from MNC containing media or blood serum were incubated at different time points. The level of histamine released into the media containing MNC in vitro and blood serum in vivo at different time-points was determined by using a competitive direct Enzyme-linked immunosorbent assay (ELISA) (CD-ELISA) (Histamine EIA Kit, Oxford Biomedical Research, UK). The level of PGI_2_ released into the media containing MNC in vitro and blood serum in vivo was determined with the measurement of 2,3-dinor-6-keto PGF 1α using an ELISA kit (EIA Kit, Cayman Chemical, USA).

### NOS Inhibitor

NO production was inhibited using L-NAME (50 mg/kg, Alexis Biochemicals, USA) in 1 ml of Phosphate buffered saline (PBS). Rats underwent stroke and at 22 hrs, MNCs were collected. At 1 hour prior to MNC or saline injection, L-NAME was administered by intraperitoneal injection (IP) while the control group received saline IP.

### NO Donor

To promote NO generation, the NO donor, MAHMA NONOate, ([Z]-1-[N-(2-aminoethyl)-N-(2-ammonioethyl) aminio] diazen-1-ium-1,2-diolate) (0.4 mg/kg, Sigma-Aldrich, USA) in 1 ml PBS was used. Rats underwent stroke and were then randomized 24 hrs later to receive saline or NONOate IV.

### Biodistribution

After stroke and bone marrow harvest at 22 hrs, MNC were labeled with Q-tracker525 (Green). At one hour prior to MNC injection, animals were administered L-NAME (50 mg/kg) or saline (N = 3 per group) IP. Ten million labeled autologous MNCs were administered IV at 24 hrs after stroke. At one hour after MNC injection, animals were sacrificed and perfused with cold PBS followed by 4% paraformaldehyde (PFA). The brain, lungs and spleen were removed and post-fixed for 24 hours and then immersed in 20% sucrose. Ten micron cryosections were generated and counterstained with 4′,6-diamidino-2-phenylindole (DAPI) (blue) prior to microscopic analysis. Q-tracker positive MNCs were quantified with NIH image software (Image J) in each experimental group on 3 sections of predefined ROIs, involving 9 fields per section randomly chosen under 400× magnification. The regions of interest were in the lungs, spleen, and in the peri-injured tissue of the brain at the level of the striatum.

### Lesion Size

Lesion size was determined in two sets of experiments. In Experiment 1, animals were randomly allocated to the following treatment groups at 1 day after stroke: (1) L-NAME IP followed by 10 million MNCs IV; (2) saline IP followed by 10 million MNCs IV; or (3) saline IP followed by saline IV (N = 8 per group). In Experiment 2, animals were randomly assigned to the following treatment groups at 1 day after stroke: (1) NONOate IV or (2) Saline. At 28 days after stroke in both sets of experiments, rats were deeply anesthetized and perfused transcardially with PBS, followed by 4% PFA in PBS. Coronal 20 um frozen sections were cut and stained with cresyl violet to measure tissue loss of the chronic infarct using the indirect method and expressed as a percentage of the contralateral hemisphere [10]. Outcomes were measured blinded to treatment groups.

### Behavioral Studies

In the same experiments above involving groups of animals that underwent lesion size, all animals underwent sensorimotor testing which was performed during the light cycle by an examiner blinded to treatment allocation. Animals were pre-tested and then tested before MCAO and on days 1, 3, 7, 14, 21, 28 post-ischemia. We used a battery of sensorimotor tests, a composite of motor, sensory, and balance tests based on the modified Neurological Severity Score (mNSS) sensitive to damage produced by our rodent ischemia model and include the following [11].

1. Cylinder [4]: The final score was calculated as (non-impaired forelimb touch+both touch)/total touch. A total of consecutive 20 touches were recorded in the 5- minutes test.
2. Circling: Animals were placed in a Plexiglas cylinder and the ratio of circling movements toward the non-paretic to the paretic side was recorded in the 2-min test.
3. Tactile Placing [12]: The number of times the rat successfully raised its forelimb to the tabletop after whiskers contacted the top surface was scored. 10 trials×2 times for each side and then the average were obtained. Value = non-impaired side – impaired side.
4. Sticker Test [11]: the time to removal of a sticker placed on the right and left forepaws was recorded within an observation time of 180 seconds. The time to removal on the right forepaw was subtracted from the time on left forepaw.
5. Beam Balance [11]: using a beam with 2-cm width and 150 cm length
6. Flexing Test [11]:Raising rat by the tail

Table 2 shows the details of different tests and scores. Outcomes were measured blinded to treatment groups.

**Table 2:** Details of different behavioral tests and scores.

Score	Circling	Cylinder	Tactile Placing	Sticker Test	Beam Balance	Flexing Test
0	<100%	0–50%	<2	<20 s	No limbs down for 60 sec	no flexion of forelimb
1	>100%	50–60%	2 to 4	20 to 60 s	Stays on beam for 60 sec but 1 limb falls	Flexion of forelimb
2	>150%	60–70%	5 to 7	60 to 90 s	Stays on beam for 60 sec but 2 limbs falls	NA
3	>200%	70–80%	8 to 10	90 to 120 s	Falls off, >40 s	NA
4	>250%	80–90%	NA	120 to 150 s	Falls off, 20–40 s	NA
5	>300%	90–100%	NA	>150 s	Falls off, <20 s	NA

### Statistics

All data are presented as Mean±SD. Statistical significance was set at p<0.05 level. Statistical analysis was performed with repeated-measures ANOVA and post hoc tests for continuous variables. Behavioral data are presented as median±SD. Statistical analysis was performed with ranked sum repeated measures testing.

## Results

### Mortality

The overall mortality was 3% and occurred in the behavioral studies (4/44 = 9%). Four animals died in the first 24 hours after stroke prior to allocation to treatment groups. As we have shown in a prior report [4], there was no mortality associated with intra-arterial delivery of MNCs.

### Cerebral Perfusion

**Figure 1:**
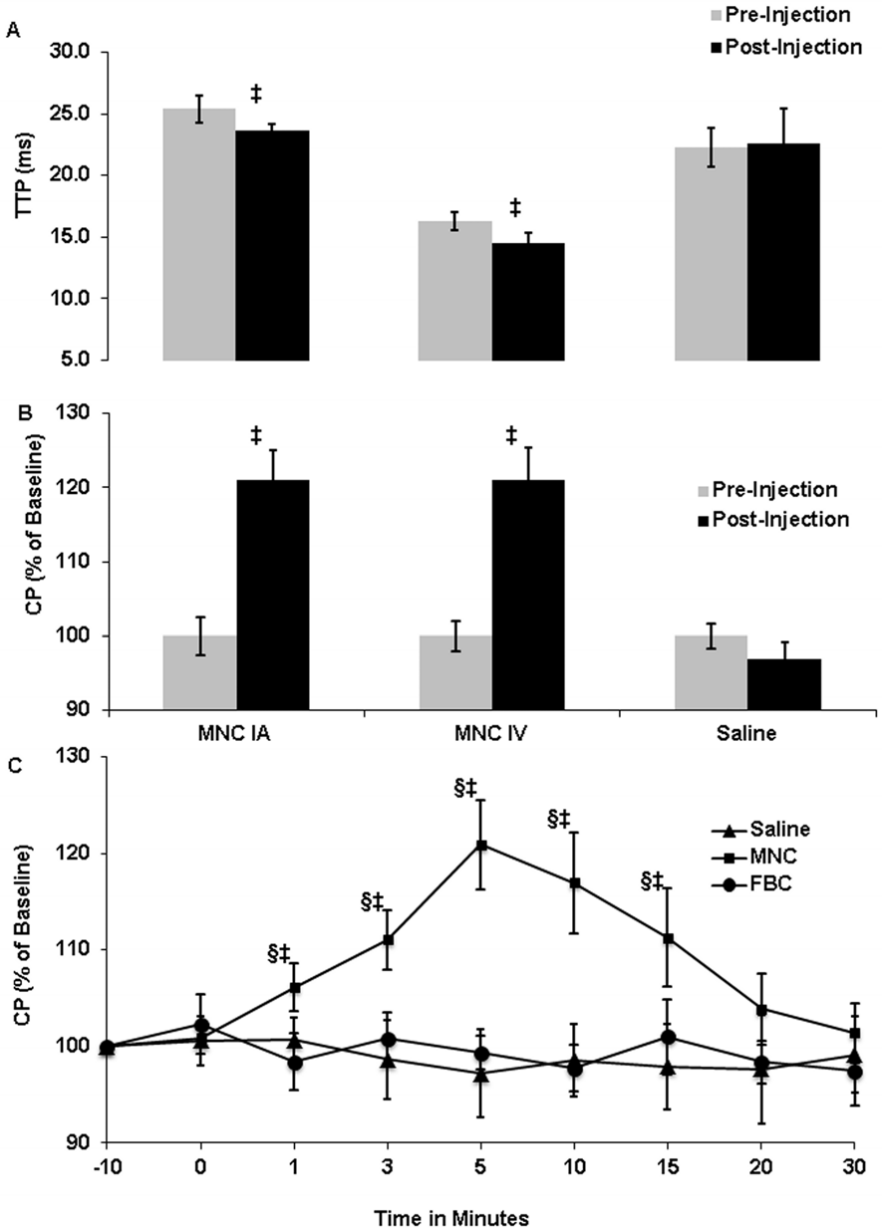
Changes in Cerebral perfusion. (A) Perfusion weighted imaging was performed in animals receiving MNC or saline at 24 hrs after MCA occlusion. There was a significant decrease (‡p<0.05) in the mean TTP in the peri-infarct region at 5 minutes after MNC infusion via IA or IV routes of delivery. There was no significant change in TTP in the saline group before and after injection. N = 4 per group. (B) Cerebral perfusion (CP) was monitored with Laser Doppler flowmetry over the peri-infarct area of animals administered MNCs (10 million cells) or saline at 24 hrs after MCA occlusion. CP was expressed after injection as a percentage compared with the baseline pre-injection value. CP increased at 5 minutes after MNC injection. There was no significant change in CP in the saline treated group. N = 6 per group. (C): Time course of changes in cerebral perfusion after IV injection of MNCs (10 million cells), FBCs (10 million), or saline. ‡p<0.05 refers to the MNC group compared to the saline group and §p<0.05 refers to the MNC group compared with the FBC group. There was significant elevation of CP after MNC infusion at 1, 3, 5, 10 and 15 minutes. There was no significant change in CP in the saline or FBC groups. ‘0’ on x-axis represents the time in minutes, just before the injection. N = 6 per group.

We found a significant decrease in TTP within the peri-infarct area at 5 minutes after MNC injection compared with saline injection. This effect was observed in both the IA and IV MNC-treated groups (p = 0.03 and p = 0.04, respectively) compared to the TTP values before injection (**Figure 1A**), indicating that these cells increase CP. To verify the observed increase in CP induced by MNCs with MRI at this time point, another group of animals were monitored using LDF over the peri-infarct area, which confirmed the findings of increased CP induced by MNC detected on MR perfusion (**Figure 1B**). We then assessed using LDF the duration of perfusion changes after injection of MNC in the peri-infarct area (**Figure 1C**) in the same experiment. CP was significantly increased by MNC from 1 to 15 minutes after injection. To assess whether the effect on perfusion is unique to MNCs, corresponding groups of animals at 24 h after stroke either received injections of saline or FBCs instead on MNCs. The FBC and saline-treated groups did not show a change in cerebral perfusion compared with baseline CP values (**Figure 1C**).

### Vasoactive Mediators

**Figure 2:**
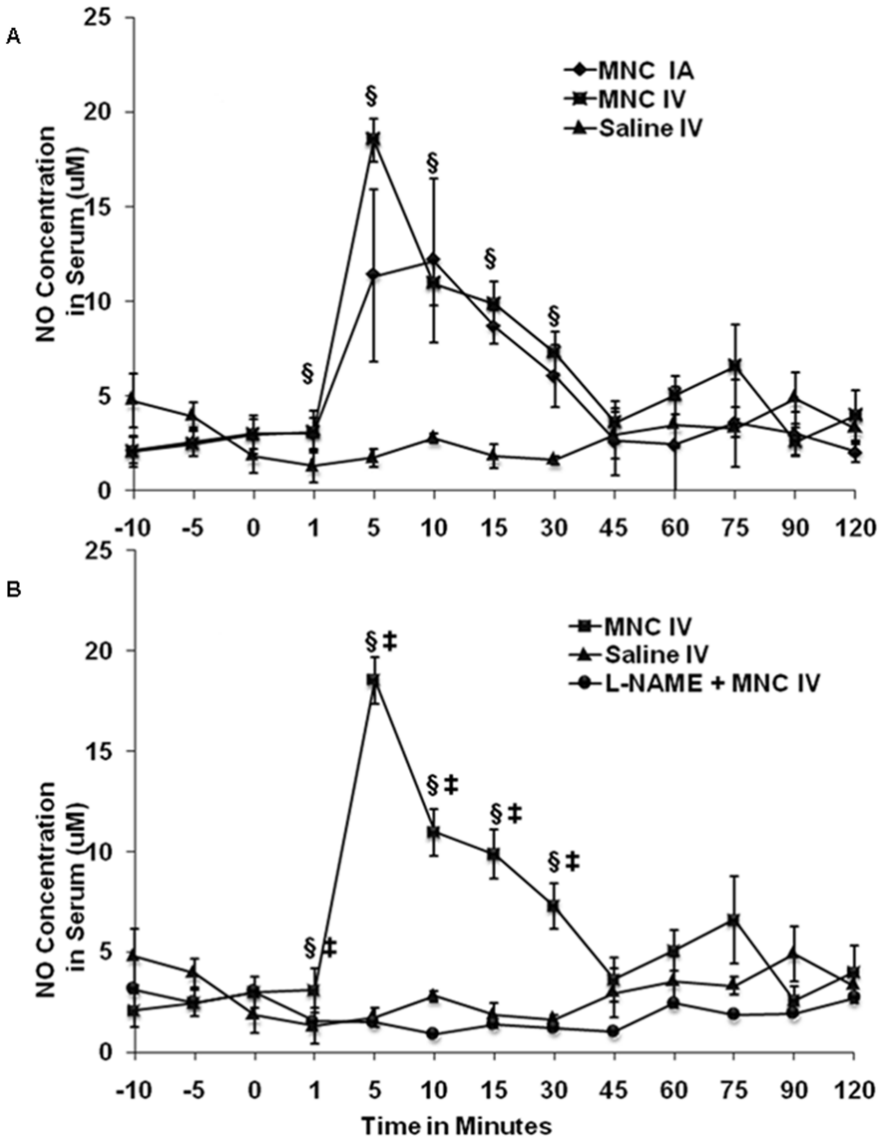
Time course of changes of NO at 24 hours after stroke. (A): Serum NO levels after saline or MNC (IV or IA) administered at 24 hrs post stroke. §p<0.05 for IA or IV MNC treated groups compared with the saline treated group. Time zero refers to the time right before cell or saline injection. N = 5 per group. (B): Serum NO levels in animals treated with L-NAME administered 60 min before MNC injection IV. §p<0.05 refers to the MNC group compared with the saline group; ‡ p<0.05 refers to the MNC group compared with the L-NAME+MNC group. N = 5 per group.

We hypothesized that MNCs may affect CP through the direct or indirect release of vasoctive mediators. To test this hypothesis, in a separate experiment, we measured the serum level of NO (**Figure 2A**), histamine, and PGI_2_ early after IA or IV injection of MNCs or saline in rats at 24 hrs after stroke. Five minutes after IV injection of MNCs, the NO level increased sharply and then decreased over the next 25 min. NO levels returned to normal at 45 min post-injection. However, there was no change in PGI_2_ or histamine levels (data not shown). To determine a causal relationship between NO and CP, in parallel experiments, we treated rats with the Nitric oxide synthase (NOS) inhibitor, L-NAME, prior to receiving MNCs. This study demonstrated that L-NAME significantly reduced the levels of serum NO compared with animals pre-treated with saline (**Figure 2B**). We then investigated whether MNCs are the source of NO by measuring its level in the media used to culture these cells. We found no detectable levels of NO present in the culture media collected at 1 min, 5 min, 15 min, 30 min, 45 min, 60 min, 2 hrs, 6 hrs, and 24 hrs after MNCs were added to the media (data not shown), suggesting that the NO release may require interaction of MNCs with the host tissue, and may involve vascular cells of animals receiving MNCs.

### MNC-induced changes in cerebral perfusion are attenuated by NOS inhibitor

**Figure 3:**
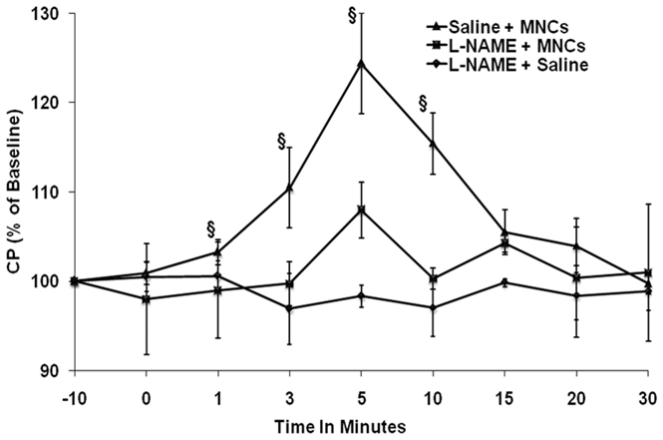
Time course of changes in cerebral perfusion after administration of MNCs at 24 hours after stroke. Changes in cerebral perfusion were measured with LDF over the peri-infarct region. Animals at 24 hrs after stroke were treated with L-NAME or saline 60 min before administration of MNCs IV. A third group of control animals received L-NAME followed by saline IV. §p<0.05 refers to the group pre-treated with saline followed by IV MNCs compared with the group pre-treated with L-NAME followed by MNCs IV. ‘0’ on the x-axis represents the time in minutes, just before the injection. N = 5 per group.

To test the hypothesis that NO contributes to the increase in perfusion induced by MNCs, animals were pre-treated with L-NAME or saline 10 minutes before IV injection of MNCs (10 million cells) at 24 h after stroke. In agreement with our data that MNCs increase CP and lead to elevated NO levels, animals pre-treated with L-NAME to inhibit NO synthesis no longer showed an increase in CP on LDF compared to saline and pre-treatment values (**Figure 3**).

### Biodistribution

**Figure 4:**
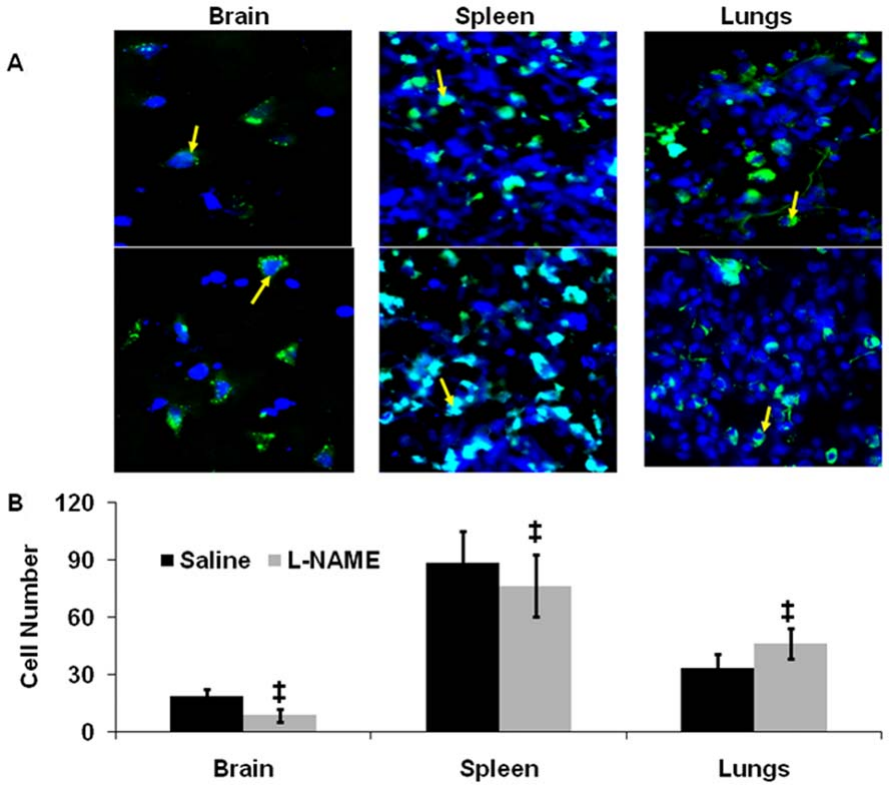
Biodistribution of MNC in brain, spleen and lungs after IV administration of MNCs(10 million) at 24 hrs after stroke. MNCs were labeled with Q-tracker(green). These animals had undergone stroke, 24 hrs later were injected with MNCs IV and then sacrificed 1 hr afterwards. Sections were prepared at 1 hr after injection and counterstained with DAPI (blue). L-NAME was injected via IP 1 hr prior to MNC administration. N = 3 per group. (A): Representative microscopic pictures illustrating MNCs (green+blue) in animals pre-treated with L-NAME (top row) or saline (bottom row). Magnification: 400×. (B): A histogram illustrating the mean number of labeled MNCs per high power field in the brain, spleen, and lungs. Animals were pre-treated with L-NAME before IV injection of MNCs or animals were pre-treated with saline followed by IV injection of MNCs (‡p<0.05).

**Figure 5:**
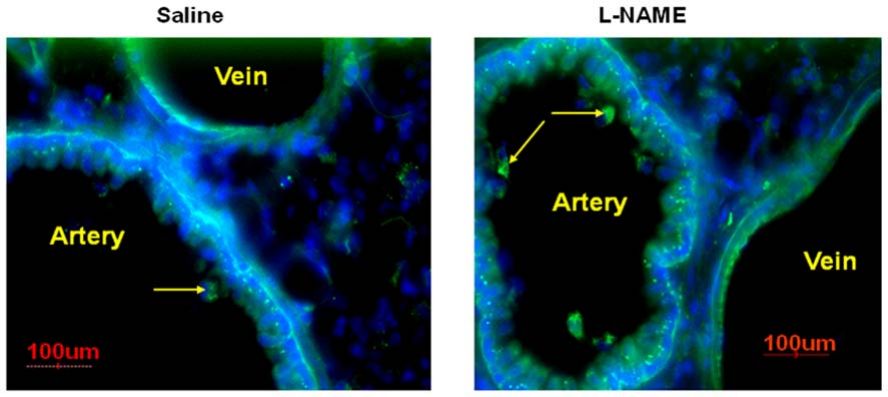
Illustration of a pulmonary artery of an animal pre-treated with L-NAME or Saline before IV MNC administration. Arrows point toward Q tracker labeled MNCs.

Assuming that NO, released in response to MNCs, underlies increased CP and vasodilation, we hypothesized that these vascular responses may be important for MNC delivery to the brain. The same mechanisms may facilitate MNC transit through the lung, which sequesters cells from the circulation [13], and enhance their delivery to the brain. To examine the relevance of NO to this process we used L-NAME, which we have shown reduces serum NO levels under the same experimental conditions. Specifically, we evaluated the biodistribution of MNCs intravenously injected into the rat at 24 hrs after stroke **(** **Figure 4** **)**. L-NAME or saline was administered 1 hr before MNC injection. L-NAME robustly reduced the number of MNCs in the peri-ischemic brain as compared to saline control (8.5±3.5 vs 18.2±4.0 p<0.01; **Figure 4B**). L-NAME also reduced the number of MNCs in the spleen (76.5±16.0 vs 99.8±16.1, p<0.01), however it increased the number of MNCs in the lungs compared to saline control (46.2±8.2 vs 33.5±7.1, p<0.05) (**Figure 4B**). Representative images of MNCs in brain, spleen and lung are shown in **(** **Figure 4A** **)**. There appeared to be more MNCs observed within the lumen and wall of the pulmonary arteries in animals that were pre-treated with L-NAME compared with saline (**Figure 5**).

### Infarct Maturation

**Figure 6:**
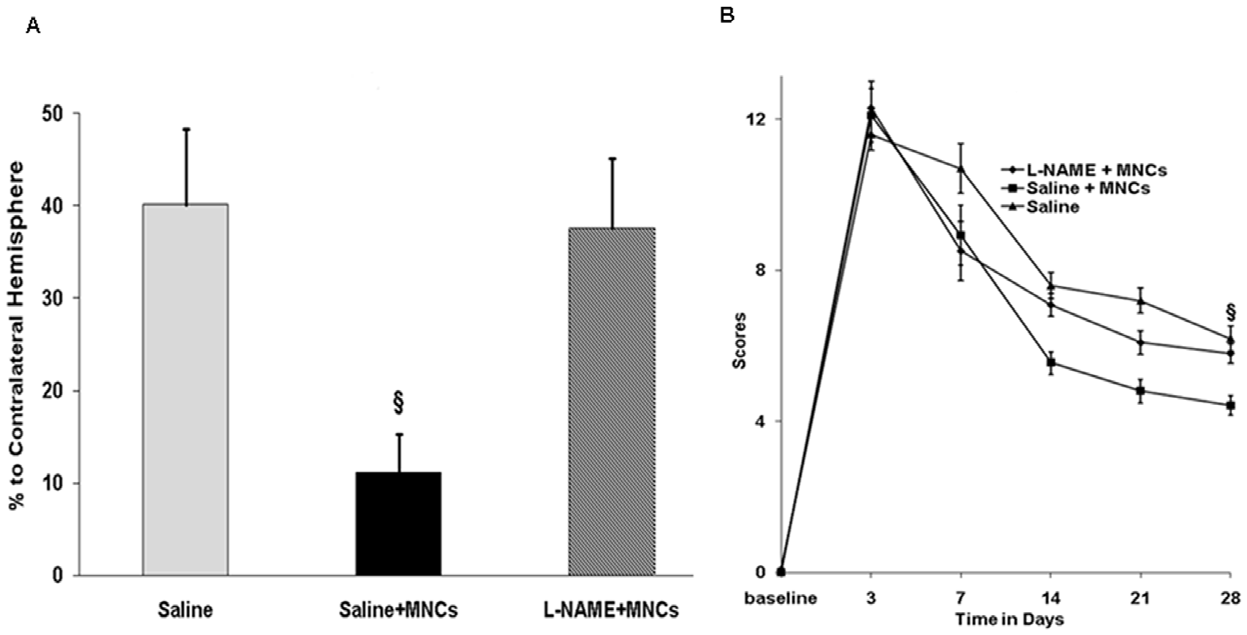
L-NAME inhibits the beneficial effect of MNCs. (A) Chronic infarct cavity at 28 days after stroke in animals treated with saline or MNCs. Animals at 24 hrs after stroke received saline followed by IV MNCs (10 million cells), or L-NAME followed by IV MNCs (10 million cells), or saline only. Lesion size was reduced by MNCs at 28 days after stroke. However, pre-treatment with L-NAME before MNC infusion abolished this reduction in lesion size (§p<0.05 compared with saline). The lesion size was calculated as a percent of the contralateral cortex N = 8 per group. (B) Neurological deficits serially evaluated after stroke in animals treated with saline IP followed by IV infusion of MNCs or L-NAME IP followed by IV infusion of MNCs. At 28 days, animals treated with saline followed by MNCs showed a significant reduction in deficits compared with animals treated with L-NAME+MNCs or compared with animals treated with saline only (§p<0.05). The x-axis represents the time in days after stroke. Baseline refers to the time point before stroke. N = 8 per group.

Although we found that MNCs increase CP transiently which is associated with NO-dependent delivery of MNCs to the brain, the question arises whether these findings have biological relevance to the pathogenesis of ischemic stroke. We addressed this question by determining if pre-treatment with L-NAME, which reduces MNC entry to the brain, ameliorates the effects of MNC on the delayed infarct maturation process after stroke, a phenomenon well established in our laboratory for this stroke model [4]. First, we confirmed that in animals treated with 10 million cells, there was a significant reduction in ischemic lesion size at 28 days, as compared with animals who received saline control (p<0.05). Next, we showed that treatment with L-NAME one hour before MNC infusion led to a loss in tissue protection (inhibition of a lesion growth) (**Figure 6A**), suggesting that NO plays an important role in mediating the cytoprotective effects of MNC in the rodent stroke model. Finally, the beneficial role for NO in the recovery process after stroke appears to be through facilitating MNC-mediated tissue preservation instead of a direct NO effect on brain tissue preservation, as the administration of the NO donor, NONOate, instead of MNC at 24 h after stroke, showed no beneficial effect on lesion volume maturation or functional deficits at 28 days (data not shown).

### Neurological Deficits

To determine if brain damage at the later stages of infarct maturation following treatment with L-NAME also affects functional recovery after stroke, we analyzed neurological deficits. In the same animals described above, we demonstrated that L-NAME in addition to increasing brain damage, also reversed the beneficial effect of MNC on neurological function, as determined at 28 days after stroke (**Figure 6B**). The administration of the NO donor, NONOate, however, at 24 h after stroke showed no effect on neurological deficits (data not shown) compared with saline controls.

## Discussion

MNCs are an attractive source for cell-based therapy because they are easily isolated from bone marrow and permit autologous use. In stroke, MNCs, when injected at 24 h after stroke, migrate to the peri-infarct region, enhance recovery and reduce post-ischemic lesion size [4], [14]. We found that MNCs transiently increase perfusion in the peri-infarct area when administered IA or IV. However, we chose an IV delivery route for most of our experiments because IV and IA were found to lead to similar effects on MR perfusion and IA delivery of saline causes an immediate decrease in LDF signal due to hemodilution in our preliminary data. The increase in CP is likely mediated, at least in part, by NO. The administration of MNCs leads to a rise in serum NO within minutes of injection and the NO synthase inhibitor, L-NAME, administered prior to MNCs attenuated both the increase in NO level and CP caused by MNCs. These results suggest a direct relationship between NO and the changes in MNC-induced perfusion. Interestingly, we found no evidence for changes in the serum of other vasoactive substances such as histamine or PGI_2_, again suggesting a prominent role for NO in this process. Finally, we have also demonstrated that the increase in CP after MNC injection is unlikely the result of vascular responses to a sudden increase in number of cell particles in the lumen as injection of an equivalent number of fibroblasts did not change CP.

Prior studies have shown that MNCs can increase cardiac perfusion weeks after administration due to an induction in angiogenesis [15] and that MNCs can increase cerebral perfusion, mediated by NO, in a global cerebral ischemia animal model [16]. Our data suggest a novel mechanism in which NO-mediated improvement in CP may assist in the arterial passage of MNCs to the peri-ischemic aspect of the brain, to a location where these cells could favorably affect secondary stroke pathogenesis and the recovery process. In agreement with this notion, we found that NO synthase inhibition, which prevents MNC-mediated NO formation and reduces CP, also adversely affected MNC's access to the brain as evidenced by a reduced number of MNCs in the ischemic brain. Since previous studies suggest that a higher number of MNCs in the brain corresponds to better functional outcome [6], it is possible that this short NO-mediated facilitation of MNC delivery to the brain could represent an important step in MNC-based therapy. Previous studies suggested that systemically injected MNCs may be filtered by the lungs [13] and in this study we found retention of more cells in the pulmonary artery with L-NAME pre-treatment. We now determine that in addition to the direct effect on MNC delivery to the brain, NO may facilitate pulmonary transit of MNCs, as inhibition of NO with an NOS inhibitor increased MNC retention in the lungs. Thus NO-mediated transit via the lungs could allow for more MNC to reach the brain to assist in damage repair after stroke. In agreement with this notion, we demonstrated that MNC brain delivery in the presence of L-NAME prevented MNCs from reducing infarct maturation and neurological impairments. These results suggest a pivotal role for NO in mediating the recovery effects of MNCs by potentially facilitating their delivery in the brain where they promote cytoprotective effects. Lastly, we also found that an NO donor, administered instead of MNCs, does not lead to better neurological outcome and attenuation of lesion size, which provides further evidence for the importance of NO signaling as a key mediator of MNC delivery to the brain and recovery from stroke.

This study has a few limitations. The use of isoflurane for the MR perfusion experiments may have been a complicating variable since isoflurane increases CBF; alternative anesthesia may need to be considered in future experiments. In addition, there are possibly other mechanisms of MNCs that may contribute to their improved long term recovery after stroke, beside NO-mediated MNC brain penetration.

In conclusion, our data for the first time demonstrate that: (1) IA and IV delivery of MNCs in older rats 24 hrs after stroke transiently increase CP within the peri-infarct region; (2) the mechanisms underlying the changes in perfusion are likely due, at least in part, to the effects of NO on cerebral vessels as MNCs cause an increase in blood NO levels and a NOS inhibitor ablates MNC effect of CP; (3) the increase in NO and CP is associated with an increased delivery of MNCs to the brain and reduced retention of MNCs in the lungs, as suggested by the reversal of this effect by a NOS inhibitor. Finally, we propose that the delivery of MNCs to the brain by NO may represent an important mechanism underlying how MNCs access the brain to mediate its effect on ischemic lesion maturation and improve recovery after stroke. Our findings raise translational considerations that perhaps an NO donor plus MNCs could enhance the efficacy of MNC-based cell therapy for stroke.
